# Integration of Gene Expression Profile Data of Human Epicardial Adipose Tissue from Coronary Artery Disease to Verification of Hub Genes and Pathways

**DOI:** 10.1155/2019/8567306

**Published:** 2019-11-27

**Authors:** Weitie Wang, Qing Liu, Yong Wang, Hulin Piao, Bo Li, Zhicheng Zhu, Dan Li, Tiance Wang, Rihao Xu, Kexiang Liu

**Affiliations:** ^1^Department of Cardiovascular Surgery of the Second Hospital of Jilin University, Ziqiang Street 218, Changchun, Jilin 130041, China; ^2^Graduate School of Medicine and Faculty of Medicine of the University of Tokyo, 7-3-1 Hongo Bunkyo-ku Tokyo, Tokyo 113-8655, Japan

## Abstract

**Background:**

This study aim to identify the core pathogenic genes and explore the potential molecular mechanisms of human coronary artery disease (CAD).

**Methodology:**

Two gene profiles of epicardial adipose tissue from CAD patients including GSE 18612 and GSE 64554 were downloaded and integrated by *R* software packages. All the coexpression of deferentially expressed genes (DEGs) were picked out and analyzed by DAVID online bioinformatic tools. In addition, the DEGs were totally typed into protein-protein interaction (PPI) networks to get the interaction data among all coexpression genes. Pictures were drawn by cytoscape software with the PPI networks data. CytoHubba were used to predict the hub genes by degree analysis. Finally all the top 10 hub genes and prediction genes in Molecular complex detection were analyzed by Gene ontology and Kyoto encyclopedia of genes and genomes pathway analysis. qRT-PCR were used to identified all the 10 hub genes.

**Results:**

The top 10 hub genes calculated by the degree method were AKT1, MYC, EGFR, ACTB, CDC42, IGF1, FGF2, CXCR4, MMP2 and LYN, which relevant with the focal adhesion pathway. Module analysis revealed that the focal adhesion was also acted an important role in CAD, which was consistence with cytoHubba. All the top 10 hub genes were verified by qRT-PCR which presented that AKT1, EGFR, CDC42, FGF2, and MMP2 were significantly decreased in epicardial adipose tissue of CAD samples (*p* < 0.05) and MYC, ACTB, IGF1, CXCR4, and LYN were significantly increased (*p* < 0.05).

**Conclusions:**

These candidate genes could be used as potential diagnostic biomarkers and therapeutic targets of CAD.

## 1. Introduction

Coronary artery disease (CAD) is a common cause of morbidity and mortality Worldwide [1]. Medical therapy and interventional or surgical techniques seem to save lots of patients with acute myocardial infarction in emergency phrase. However, long term outcomes remain unsatisfactory [2]. Thus further understanding of the etiology may provide potential diagnosis and therapeutic method for CAD. Recently, many reports present that epicardial adipose tissue (EAT) plays an important role in the progression of many disease by secretion various bioactive molecules [3]. In addition, finding also shows that the changing state of EAT will significantly affect the cardiac function and increase cardiovascular risk in human beings [4, 5].

CAD is a kind of coronary artery disease and most studies aim to research through the blood sample. However, EAT is found to have relevant with the heart because it is a type of visceral fat depot. The EAT widely distributes between the pericardium and myocardium which serves with metabolically activation [6]. Owing to its special location and close proximity to coronary vasculature, comparison of different express gene between EAT[7] and subcutaneous adipose tissue (SAT) may provide important information about the state of the coronary artery and maybe have potential for diagnosis and therapy for human CAD.

mRNAs have been reported to participate in the regulation of pathophysiological conditions of cardiovascular disease (CVD) [8]. However, the existing results of mRNAs profiles of CAD can not identify the core pathogenic genes and the potential molecular mechanisms of human CAD [9–11]. Therefore, we downloaded the two mRNAs profiles data and screened out the co-expression differentially expressed genes (DEGs) between GSE 18612 and GSE 64554. After analyzing by biomathematical online tools and software, 10 hub genes were finally identified which could serve as new biomarkers and therapeutic targets for human CAD.

## 2. Materials and Methods

### 2.1. Microarray Analysis

This study was conducted in accordance with the Declaration of Helsinki and was approved by the Ethics Committee of the Second Hospital of Jilin University. The two mRNA profiles were searched by CAD and EAT as well as human in gene expression omnibus (GEO) database (http://www.ncbi.nlm.nih.gov/geo). All the data were provided from GPL96 platform. GSE 18612 contained 7 EAT from CAD patients and 6 SAT from noncoronary artery disease (NC) and there were 13 EAT from CAD patients and 10 SAT from NC patients in GSE 64554. All the data were analyzed with GEO2R online tool with two classifications including EAT and SAT.

### 2.2. Data Processing and Identifcation of DEGs

Downregulated and upregulated genes were selected in each profile with *p* < 0.05 and fold change >1.2 or < −1.2. *R* sofware including affy package [12]and limma package [13] were used to identify the final co-expression DEGs.

### 2.3. Gene Ontology and Pathway Enrichment Analyses

All DEGs and hub genes as well as predict genes in Molecular Complex Detection (MCODE) were analysis by DAVID (the Database for Annotation, Visualization, and Integrated Discovery) online bio-informatics database [14]. Gene ontology (GO) enrichment including cellular component (CC), biological processes (BP) and molecular function (MF) were acquired from DAVID. Pathway analysis of all DEGs was analysised by KEGG pathways tool. All the results were selected with gene count >2 and *p* < 0.05.

### 2.4. Integration of Protein-Protein Interaction (PPI) Network Analysis

The DEGs were totally typed into STRING (https://string-db.org/cgi/input.pl) database [15] to get PPI networks and the interaction data among all co-expression genes were picked with a significant confidence score >0.9. Then the interaction data were typed into the Cytoscape software [16] to structure a PPI network. Based on the above data, we used MCODE [17], a built-in APP in Cytoscape software, to analyze the interactive relationship of the DEGs encoding proteins and screen hub gene. The parameters of network scoring and cluster finding were set as follows: find clusters = in whole network, degree cutoff = 2, cluster finding = haircut, node score cutoff = 0.2, k-core = 2, and max depth = 100. The parameters of cytoHubba were set as follows: Hubba nodes = top 10 nodes ranked by degree, display options = check the first-stage nodes, display the shortest path and display the expanded sub network.

### 2.5. Quantitative Reverse Transcription-PCR (qRT-PCR) Validation and Statistical Analysis

qRT-PCR was used to verify the top 10 hug genes. PrimeScript RT reagent Kit with gDNA Eraser (TaKaRa, Japan) was used to provide the process of RNA reverse-transcribed to cDNA. Primers were designed from Primer-BLAST (https://www.ncbi.nlm.nih.gov/tools/primer-blast) and listed in Table 1. QuantStudio 7 Flex real-time PCR system (Applied Biosystems, Carlsbad, CA, USA) was used [18, 19] and samples were normalized to GAPDH. All the operations were according to the manufacturer's instructions. The relative expression levels of each gene were calculated using 2^−ΔΔCt^ methods.

## 3. Results

### 3.1. Identification of DEGs in the CAD and NC Groups

All of 20 EAT from CAD patients and 16 SAT from NC patients were analyzed with *R* software with a threshold setting at *p* < 0.05 and fold change >1.2. There were 2617 and 2192 DEGs in GSE 18612 and GSE 64554 mRNAs profiles, respectively. Finally, all 179 co-expression DEGs were selected through integrating analysis, which included 105 downregulated and 74 upregulated DEGs in EAT from CAD samples.

### 3.2. GO Functional Enrichment Analysis

**Figure 1:**
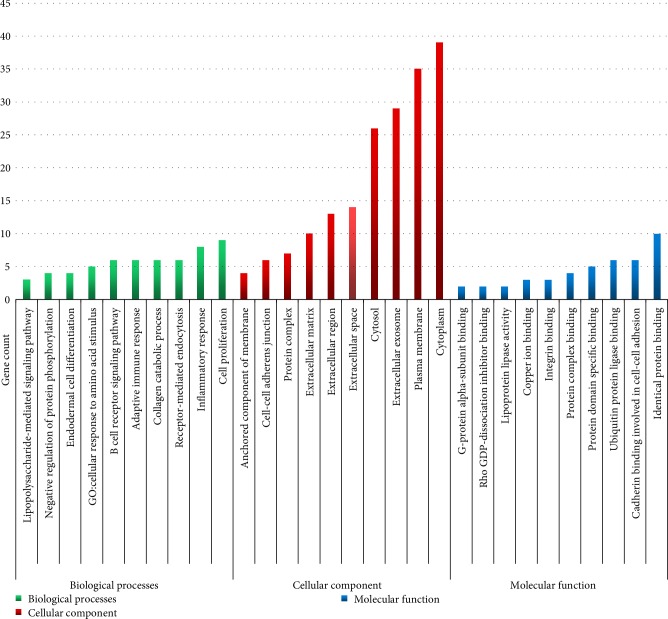
Gene Ontology analysis classified the differentially expressed genes into 3 groups:****molecular function, biological process, and cellular component.

All DEGs including downregulated and upregulated co-expression genes were typed into DAVID online tool respectively. The functions of GO analysis were divided into three classifications: BP, CC, and MF (Figure 1). As shown in Figure 1 and Table 2, in the biological processes group, the upregulated DEGs were mainly enriched in B cell receptor signaling pathway, cellular response to drug, adaptive immune response, inflammatory response, and negative regulation of protein phosphorylation, and the downregulated DEGs were mainly enriched in collagen catabolic process, cellular response to amino acid stimulus, endodermal cell differentiation and cell proliferation. In the cellular component group, the upregulated DEGs were mainly enriched in plasma membrane, cytosol, extracellular space, anchored component of membrane and extracellular region, and the downregulated DEGs were mainly enriched in extracellular matrix, extracellular exosome, protein complex, cytoplasm and cell-cell adherens junction. In the molecular function group, the upregulated DEGs were mainly enriched in identical protein binding, copper ion binding, protein complex binding, integrin binding and G-protein alpha-subunit binding, and the downregulated DEGs were mainly enriched in Rho GDP-dissociation inhibitor binding, ubiquitin protein ligase binding, cadherin binding involved in cell-cell adhesion, protein domain specific binding and lipoprotein lipase activity.

### 3.3. Signaling Pathway Analysis

**Figure 2:**
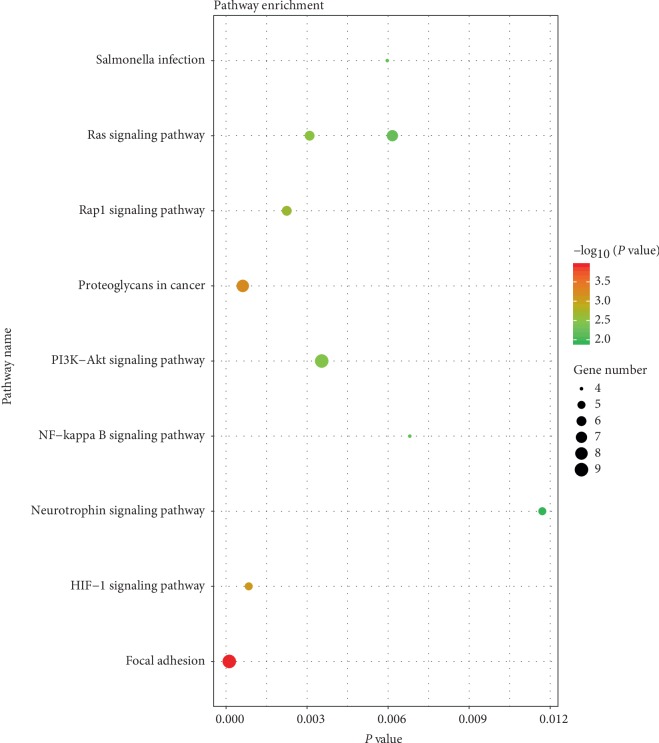
Kyoto encyclopedia of genes and genomes enrichment analysis of the pathways. The gradual color represents the *P* value; the size of the black spots represents the gene number.

KEGG pathway analysis on DAVID online tool indicated that upregulated genes were mainly enriched in HIF-1 signaling pathway, Rap1 signaling pathway, Ras signaling pathway, Salmonella infection and NF-kappa B signaling pathway. The downregulated genes were mainly enriched in Focal adhesion, Proteoglycans in cancer, PI3K-Akt signaling pathway, Ras signaling pathway, and Neurotrophin signaling pathway (Figure 2).

### 3.4. PPI Network and Modular Analysis

**Figure 3:**
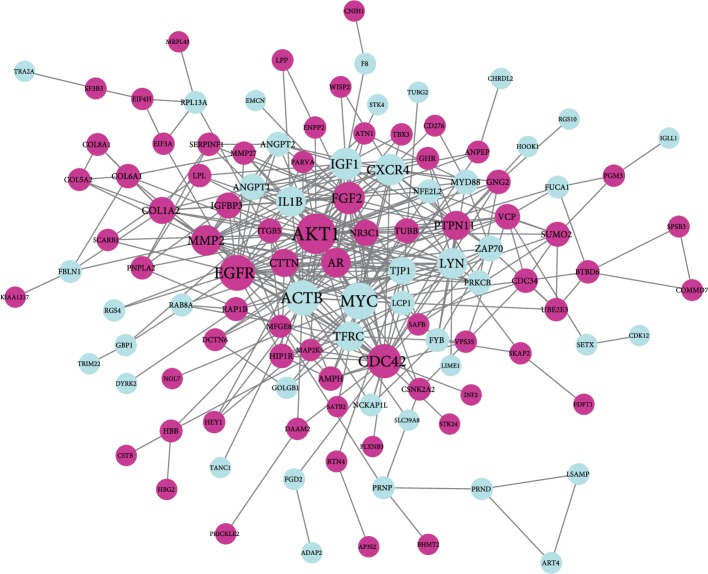
PPI network constructed with the differentially expressed genes. Blue nodes represent upregulated genes, purple nodes represent downregulated gene.

**Figure 4:**
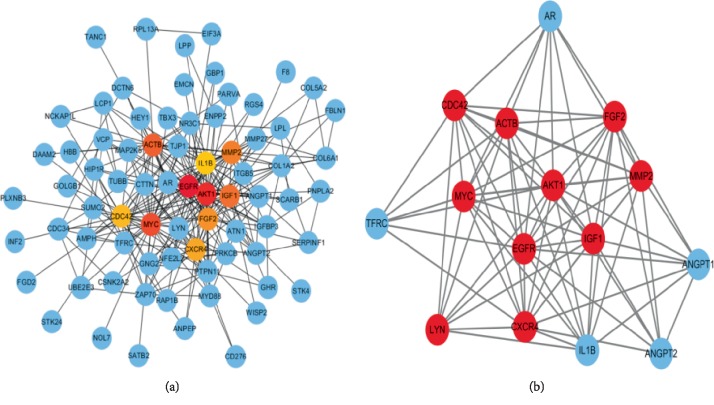
The top 10 hug gene analysis by cytoHubba (a). The gradual color represents the degree score. The most significance modules (b). Red nodes represent hug gene analysis by cytoHubba.

STRING database presented a result with 178 nodes and 363 edges (Figure 3) after analysis of all the DEGs. The nodes and edges were put into the cytoscape software in order to acquire a PPI network. CytoHubba analysis section showed 10 hub genes, including AKT1, MYC, EGFR, ACTB, CDC42, IGF1, FGF2, CXCR4, MMP2, and LYN after degree calculation. In these 10 hub genes, AKT1 presented with the highest degree (degree = 38). The cytoscape plugin MCODE showed the top three modules with scores of 11.429, 3.333, and 3.000 (Figure 4). MCODE 1 contained 15 nodes including IL1B, AKT1, ANGPT2, MYC, LYN, FGF2, EGFR, TFRC, AR, ACTB, MMP2, IGF1, CDC42, CXCR4, and ANGPT1 with 80 edges. Then, all the 15 prediction genes in module 1 were analyzed by GO and KEGG analysis, which mainly aimed to Focal adhesion, MAPK signaling pathway, Salmonella infection, Adherens junction and Regulation of actin cytoskeleton. Module 2 was mainly associated with the Ubiquitin mediated proteolysis and Protein processing in endoplasmic reticulum. Module 3 was mainly associated with Protein digestion and absorption, ECM-receptor interaction, Focal adhesion and PI3K-Akt signaling pathway.

**Figure 5:**
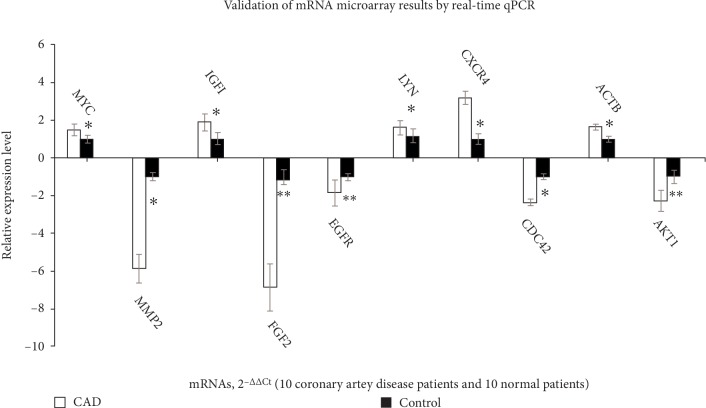
Validation of the mRNA microarray results by real-time qPCR. mRNA microarray results were verified by real-time qPCR between the CAD group (*n* = 10) and the control group (*n* = 10). All samples were normalized to the expression of GAPDH, and the relative expression levels of each gene were analyzed using the 2^−Δ Δ Ct^method. ^*∗*^*P* < 0.05, ^*∗∗*^*P* < 0.01.

All the top 10 hub gene were verified by qRT-PCR which presented that AKT1, EGFR, CDC42, FGF2 and MMP2 were significantly decreased in EAT of CAD samples (*p* < 0.05) and MYC, ACTB, IGF1, CXCR4, and LYN were significantly increased (*p* < 0.05) (Figure 5). All validation data were consistent with the microarray data and analytical results in this study.

## 4. Discussion

Adipose tissue has been thought to act as an endocrine organ to participate and regulate the inflammatory process of CVD through paracrine or endocrine pathway. Recent research points that EAT, an atypical fat depot surrounding the heart, plays essential roles in CAD not only highly metabolic paracrine and endocrine functions, but also by its blood supply from coronary circulation [20]. Therefore some changes of EAT may directly indicate the different state of the heart as they supplies from the same coronary blood. In the meantime, studies have points that metabolic function of EAT may participate the initiation and progression of atherosclerosis [21]. So researching on the EAT can be able to provide a promising therapeutic target for the treatment of CVD including atherosclerosis.

With the fast development of sequencing technology, finding the changing genes between disease and normal tissue has become convenient. Up to now, there are many mRNAs profiles have been carried out and a lot of different genes have been detected between EAT and SAT from CAD patients [9–11]. mRNAs act as protein-coding genes, are believed to play an important role on modulating the metabolic function of adipose tissue and CAD. However, the initially change and the core pathogenic gene of CAD are not identified and the key pathogenesis of CAD has not been confirmed until now.

As different expression profiles of CAD provided various results with each other, we integrated the mRNAs profiles and analysised the co-expression DEGs. In this study, 105 downregulated DEGs and 74 upregulated DEGs were identified after integrating profile datasets. Similar to other studies [22, 23], these DEGs were dealt with GO and pathway analysis and a lot of KEGG pathways were found. However, the hub gene in CAD and the most relevant pathway and mechanism of CAD could not be identified. Therefore, we used more bioinformation tools such as PPI Network online tool, cytoscape analysis with cytoHubba and MCODE analysis for further analyses. The cytoHubba analysis showed that the top ten hub genes were AKT1, MYC, EGFR, ACTB, CDC42, IGF1, FGF2, CXCR4, MMP2, and LYN. DAVID on line analysis tool predicted that these genes were highly relevant with the focal adhesion pathway in CAD. In addition, the MCODE provided another analysis of these DEGs. The top 1 MCODE contained 15 nodes including IL1B, AKT1, ANGPT2, MYC, LYN, FGF2, EGFR, TFRC, AR, ACTB, MMP2, IGF1, CDC42, CXCR4, ANGPT1 and 80 edges. These 15 nodes also indicated that Focal adhesion might act as the most relevant pathway after analysis by DAVID. Even better, the predicted genes in top 1 MCODE also contained all the top 10 hug genes. Therefore, we aimed to analyze the hub gene AKT1 and the Focal adhesion in CAD.

Akt kinase has been identified as one of the member of AGC kinases (AMP/GMP kinase and PKC subfamily of proteins), which is reported to play an important role in cell proliferation, migration and growth [24]. Although Akt1, Akt2, and Akt3 are the 3 isoforms of Akt, they play different functions in various cell environment. Recent study shows that Akt acts critical roles in the CVD including atherosclerosis [25]. The reduction of endothelial cell migration was found when Akt phosphorylation influenced by oxidized low density lipoprotein (LDL) [26]. In addition, inhibition of Akt in macrophages was also presented with reducing atherosclerosis [27]through decreasing in proinflammation and immune cell migration. Recent study also presented that inhibition of vascular endothelial growth factor (VEGF) through inducing Akt phosphorylation pathway might protect arteries from atherosclerosis through changing the peroxisome proliferator-activated receptors (PPARs). Therefore, the toppest hub gene Akt was believed to serve as a hub gene in the initial and progressive pathological in atherosclerosis.

Akt has been reported to activate or over expression with focal adhesion kinase (FAK) pathway signal. Reports show that interaction between FAK and Akt1 is found in many cancers. This interaction is always needed for subsequent FAK autophosphorylation, activation, and translocation to the focal adhesion complex (FAC) [28]. FAK was reported to participate in regulating cell adhesion, mobility and proliferation during many cell process. The focal adhesion signaling had provided insight into the role of FAK in cardiomyocyte signal transduction and had been reported to contribute to myocardial remodeling and the progression to heart failure [29]. In addition, FAK was also reported taking part in regulating the function of endothelial barrier [30]. Low expression of FAK in endothelial cells would result in formation of a tighter endothelial monolayer through raising cell attachment and connection. In addition, disruption of FAC would also lead to Ado/HC-induced endothelial apoptosis [31] and lipid would deposition following endothelial cell damage. Study also pointed that FAK played a role in reduction of lipopolysaccharide-induced inflammation injury through inactivation of the Wnt and NF-kappaB pathways [32] and inflammation injury was also an important factor during CAD. So focal adhesion signaling might act an important role in vascular endothelial dysfunction and pro-inflammatory reactions, and might be a core change during CAD.

In summary, by integration of different high-throughput sequencing profiles and data processing as well as hub genes, these genes may have the potential to be used as drug targets and diagnostic markers of CAD. The focal adhesion maybe the key pathway in CAD. However, there are still some limitations: further experimental studies with larger sample sizes are needed to confirm the role of the focal adhesion pathway in CAD.

## Figures and Tables

**Table 1:** The primers of the top 10 hub genes.

Gene name	Forward primer	Reverse primer
AKT1	AGCGACGTGGCTATTGTGAAG	GCCATCATTCTTGAGGAGGAAGT
MYC	GGCTCCTGGCAAAAGGTCA	CTGCGTAGTTGTGCTGATGT
EGFR	AGGCACGAGTAACAAGCTCAC	ATGAGGACATAACCAGCCACC
ACTB	CATGTACGTTGCTATCCAGGC	CTCCTTAATGTCACGCACGAT
CDC42	CCATCGGAATATGTACCGACTG	CTCAGCGGTCGTAATCTGTCA
IGF1	GCTCTTCAGTTCGTGTGTGGA	GCCTCCTTAGATCACAGCTCC
FGF2	AGAAGAGCGACCCTCACATCA	CGGTTAGCACACACTCCTTTG
CXCR4	ACTACACCGAGGAAATGGGCT	CCCACAATGCCAGTTAAGAAGA
MMP2	TACAGGATCATTGGCTACACACC	GGTCACATCGCTCCAGACT
LYN	GCTTTTGGCACCAGGAAATAGC	TCATGTCGCTGATACAGGGAA
GAPDH	CGGACCAATACGACCAAATCCG	AGCCACATCGCTCAGACACC

**Table 2:** The significantly enriched analysis of differentially expressed genes in coronary artery disease.

Expression	Category	Term	Description	Gene count	*P*-value
UP-DEGs	BP	B cell receptor signaling pathway	GO:0050853	6	2.74E-06
	BP	Adaptive immune response	GO:0002250	6	3.55E-04
	BP	GO:inflammatory response	GO:0006954	8	9.15E-04
	BP	Negative regulation of protein phosphorylation	GO:0001933	4	2.02E-03
	BP	Lipopolysaccharide-mediated signaling pathway	GO:0031663	3	7.62E-03
	CC	Plasma membrane	GO:0005886	35	5.28E-06
	CC	Cytosol	GO:0005829	26	7.67E-04
	CC	Extracellular space	GO:0005615	14	2.47E-03
	CC	Anchored component of membrane	GO:0031225	4	1.05E-02
	CC	Extracellular region	GO:0005576	13	2.56E-02
	MF	Identical protein binding	GO:0042802	10	2.44E-03
	MF	Copper ion binding	GO:0005507	3	2.02E-02
	MF	Protein complex binding	GO:0032403	4	4.68E-02
	MF	Integrin binding	GO:0005178	3	6.36E-02
	MF	G-protein alpha-subunit binding	GO:0001965	2	7.18E-02
DOWN-DEGs	BP	Collagen catabolic process	GO:0030574	6	3.55E-05
	BP	GO:cellular response to amino acid stimulus	GO:0071230	5	1.60E-04
	BP	Endodermal cell differentiation	GO:0035987	4	5.09E-04
	BP	Cell proliferation	GO:0008283	9	1.35E-03
	BP	Receptor-mediated endocytosis	GO:0006898	6	4.68E-03
	CC	Extracellular matrix	GO:0031012	10	3.94E-05
	CC	Extracellular exosome	GO:0070062	29	1.09E-03
	CC	Protein complex	GO:0043234	7	2.71E-02
	CC	Cytoplasm	GO:0005737	39	3.18E-02
	CC	Cell-cell adherens junction	GO:0005913	6	3.39E-02
	MF	Rho GDP-dissociation inhibitor binding	GO:0051022	2	2.21E-02
	MF	Ubiquitin protein ligase binding	GO:0031625	6	2.21E-02
	MF	Cadherin binding involved in cell-cell adhesion	GO:0098641	6	2.30E-02
	MF	Protein domain specific binding	GO:0019904	5	2.89E-02
	MF	Lipoprotein lipase activity	GO:0004465	2	4.37E-02

## Data Availability

The data used to support the findings of this study are available from the corresponding author upon request.

## Abbreviations

CAD: Coronary artery disease
EAT: Epicardial adipose tissue
SAT: Subcutaneous adipose tissue
DEGs: The differentially expressed genes
GEO: The gene expression omnibus
NC: Noncoronary artery disease
GO: Gene ontology;
KEGG: Kyoto encyclopedia of genes and genomes
CC: Cellular component
BP: Biological processes
MF: Molecular function
PPI: Protein-protein interaction
MCODE: Molecular complex detection
qRT-PCR: Quantitative reverse transcription-PCR
CVD: Cardiovascular disease
AGC kinases: AMP/GMP kinase and PKC subfamily of proteins
LDL: Low density lipoprotein
VEGF: Vascular endothelial growth factor
PPARs: Peroxisome proliferator-activated receptors
FAK: Focal adhesion kinase
FAC: Focal adhesion complexes
SDF1: Stromal derived factor 1
EPCs: Endothelial progenitor cells
GHSR: Growth hormone secretagogue receptor.

## Additional Points

The elements in the manuscript have not been published or are under consideration for publication elsewhere.
